# GlycoAudit: structural joinability and annotation degree shape glycan–agent relation ranking

**DOI:** 10.1093/bioadv/vbag308

**Published:** 2026-10-20

**Authors:** Minqi Chen, Minrui Chen, Guanjie Zou, Lunxu Liu

**Affiliations:** Department of Thoracic Surgery and Institute of Thoracic Oncology, West China Hospital, Sichuan University, Chengdu, Sichuan 610041, China; Western China Collaborative Innovation Center for Early Diagnosis and Multidisciplinary Therapy of Lung Cancer, Sichuan University, Chengdu, Sichuan 610041, China; School of Information Science and Electrical Engineering, Kyushu University, Fukuoka 819-0395, Japan; Department of Computational Biology and Medical Science, The University of Tokyo, Kashiwa, 277-8563, Japan; RIKEN Center for Advanced Intelligence Project, Tokyo 103-0027, Japan; Department of Thoracic Surgery and Institute of Thoracic Oncology, West China Hospital, Sichuan University, Chengdu, Sichuan 610041, China; Western China Collaborative Innovation Center for Early Diagnosis and Multidisciplinary Therapy of Lung Cancer, Sichuan University, Chengdu, Sichuan 610041, China

## Abstract

**Motivation:**

Benchmark scores can be misleading when identifier resolution, structural availability, and incomplete interaction annotation determine which glycans can be evaluated. GlycoAudit audits this evidence-to-structure path before model comparison.

**Results:**

A strict accession, branch, and sequence contract retained 44 of 173 resolved SugarBind glycans (25.4%) and excluded 129 (74.6%). The joined domain was substantially more densely annotated. Under leakage-controlled held-out-positive ranking, the Web3 Unique Representation of Carbohydrate Structures (WURCS) exceeded the evaluated global structural summaries. Yet a leave-one-agent-out annotation-degree control achieved higher mean reciprocal rank (0.491 versus 0.381) and higher known-positive average precision ($AP_{\text{known}}$), 0.489 versus 0.373, whereas WURCS achieved higher Recall@5 (0.688 versus 0.456). These metric-dependent reversals show that structural joinability, annotation degree, candidate construction, and evaluation choice jointly shape apparent benchmark performance. The release evaluates positive-relation ranking, not confirmed non-binding classification or cross-source transfer.

**Availability and implementation:**

GlycoAudit makes evidence, joining decisions, exclusions, candidates, folds, controls, and machine-readable results inspectable in the public GlycoAudit repository.

## 1 Introduction

Glycan recognition depends on branching, linkage, stereochemistry, conformational variability, epitope exposure, and biological context. Local and 3D geometry can therefore be essential to structure–function interpretation, while deposited structures often represent only part of a glycan’s accessible conformational space (Ives *et al.* 2024, Nieto-Fabregat *et al.* 2024, Thomès *et al.* 2025). Interaction evidence and structural descriptions are distributed across heterogeneous resources rather than recorded under one uniform experimental and identifier contract.

This heterogeneity creates a hidden benchmark problem before model comparison begins. A literature-supported relation may lack an exactly joinable structure, and an absent relation is not a verified negative. If structural availability is associated with annotation density, joining changes not only sample size but also the population and estimand being evaluated. Positive–unlabeled methodology makes the distinction between recorded positives and unlabeled pairs explicit (Elkan and Noto 2008, Bekker and Davis 2020); leakage-aware evaluation further requires learning and prediction information to remain separated (Kaufman *et al.* 2012).

GlyTouCan and the Web3 Unique Representation of Carbohydrate Structures (WURCS) provide stable glycan identifiers and sequence notation, whereas SugarBind, glycan arrays, GlyCosmos, and GlycoPOST provide complementary evidence, repository, and provenance functions (Blixt *et al.* 2004, Tanaka *et al.* 2014, Mariethoz *et al.* 2016, Fujita *et al.* 2021, Watanabe *et al.* 2021, Lee *et al.* 2025). SweetNet, GlyNet, glycowork, GLAMOUR, GlycanML, and GlycoGym support sequence-, graph-, property-, or interaction-oriented glycan learning (Burkholz *et al.* 2021, Thomès *et al.* 2021, Carpenter *et al.* 2022, Mohapatra *et al.* 2022, Joeres and Bojar 2025, Xu *et al.* 2025). GlycoShape and GlyContact extend 3D structure retrieval and analysis (Ives *et al.* 2024, Thomès *et al.* 2025). These contributions do not remove the need for an auditable contract linking evidence records to the exact structures and candidates used for evaluation.

GlycoAudit makes that contract inspectable through evidence provenance, identifier resolution, strict structure joining, exclusions, fixed candidates, predefined folds, and non-molecular controls. Two empirical findings dominate the present study: strict structural joining retains an annotation-enriched subset, and annotation degree changes which ranking method appears strongest across metrics. WURCS, the Structural Shape Vector (SSV), and the Glycan Contact Vector (GCV) are evaluated as diagnostic molecular baselines; the paper’s central contribution is the benchmark audit that precedes their comparison.

## 2 Methods

### 2.1 Evidence sources and evidence states

All analyses use the dated reconstruction_2026_08_04 release assembled from archived public snapshots under declared source and join contracts (Fig. 1). SugarBind evidence was obtained through the GlyCosmos SugarBind graph and represented as one literature-curated positive assertion per interaction–ligand record (Mariethoz *et al.* 2016). “Agent” denotes the SugarBind database identifier, rather than an inferred unique biological entity. The release distinguishes positive, reported non-binding, inconclusive, unmeasured, and insufficient-context evidence; missing pairs remain unlabeled.

**Figure 1:**
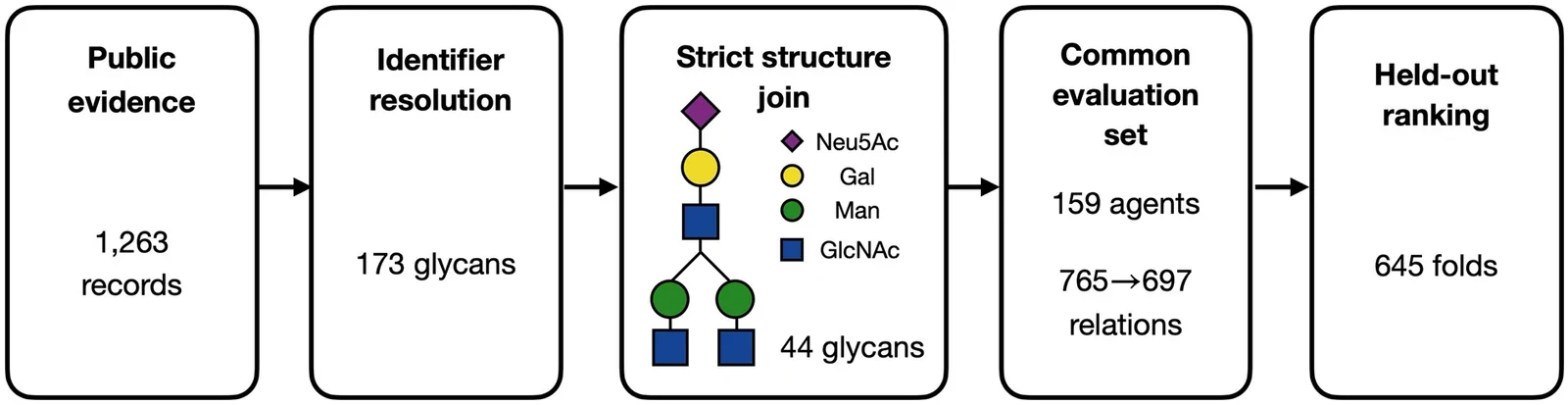
Audited benchmark workflow. Public SugarBind evidence was mapped to GlyTouCan accessions, evaluated against the strict GlycoShape structure contract, restricted to the common SSV/GCV/WURCS feature set, and assessed with predefined held-out-positive folds. Counts are records at acquisition, unique resolved and joined glycans at the next two stages, 765 canonical joined relations and 697 relations retained for agents with sufficient fold support, and agent-folds in the ranking analysis. The release also retains all exclusions, evidence states, sensitivity analyses, and automated consistency checks.

The Consortium for Functional Glycomics (CFG) catalog contributes experiment metadata but not quantitative labels (Blixt *et al.* 2004). The reconstructed catalog does not contain assay-resolved relative fluorescence units (RFU) or the historical experiment-specific positivity rules. It therefore supports provenance and scope auditing, but not binding-versus-non-binding classification or cross-source transfer in the present release.

### 2.2 Identifier resolution and strict structure joining

Stable GlyTouCan accessions and WURCS strings were retrieved for mapped SugarBind ligands (Tanaka *et al.* 2014, Fujita *et al.* 2021). GlycoShape metadata were resolved to the requested alpha or beta branch, after which the corresponding level-1 multi-model structure was accepted only when its accession, branch, Protein Data Bank (PDB) annotation, and normalized WURCS sequence agreed (Ives *et al.* 2024). Every evidence record retained its mapping status, structure decision, and failure category. Coverage is reported with its record-level or unique-glycan denominator.

### 2.3 Joinability-selection analysis

The selection analysis compared the 44 resolved accessions satisfying the strict structure contract with 129 resolved accessions that did not. Five unresolved source identifiers were described separately and excluded from group comparisons. Variables were WURCS sequence length, evidence records per glycan, canonical relations per glycan, and distinct SugarBind agent identifiers per glycan. Continuous variables were summarized by medians, interquartile ranges, means, and standard deviations. The standardized mean difference was the difference in group means divided by the pooled standard deviation. Mean-difference intervals used 10 000 nonparametric bootstrap resamples with seed 20260831, and two-sided Mann–Whitney tests compared distributions. Holm correction was applied across the four continuous-variable tests. Failure categories were tabulated descriptively.

### 2.4 Representation construction

SSV summarizes global size and geometry for each deposited coordinate model: atom and residue counts, radius of gyration, maximum atomic separation, compactness, graph-based branching and terminal-residue counts, and local atomic exposure. Glycan-level features are the mean and standard deviation across the five deposited models.

GCV summarizes a residue-center contact graph. Its scientific quantities include contacts per residue, long-range contacts, residue-neighbor contact counts, variation in pairwise residue-center distances, the graph spectral gap, the relative balance of high- and low-degree residues, and maximum sequence separation among contacting residues. The implementation default of 4.5 Å was retained as a reference cutoff rather than a pre-result or mechanistically privileged choice; 4.0, 4.5, and 5.0 Å are therefore reported as sensitivity settings. Exact feature definitions and legacy software aliases are provided in the Data Dictionary, available as supplementary data at *Bioinformatics Advances* online.

The WURCS baseline maps character *n*-grams of length 3–5 into 4096 deterministic hash buckets. Within each fold, term frequency–inverse document frequency (TF–IDF) is fitted after excluding held-out positive glycan rows, followed by $L_{2}$ normalization. WURCS, SSV, and GCV are compared on the same 44-glycan candidate universe.

### 2.5 Candidate sets and held-out-positive evaluation

Agents with at least two positive relations in the common universe were included. The fixed candidate set contains all 44 glycans for each of 159 evaluable agent identifiers. Every positive relation was assigned to one of up to five predefined agent-stratified folds with seed 42. For agent *a* and fold *f*, the reference vector was the mean of the training-positive feature vectors,


$$p_{a,f}=|P_{a,f}^{\text{train}}{|}^{-1}\sum_{g\in P_{a,f}^{\text{train}}}x_{g}.$$

Training positives were removed from the ranked candidate set, and the remaining glycans were ranked by cosine similarity to $p_{a,f}$. Median imputation and standardization for SSV/GCV, or IDF for WURCS, were fitted without held-out positive rows. This prevents relation-level reuse between reference construction and evaluation.

The reported metrics are mean reciprocal rank (MRR), Recall@5, and $AP_{\text{known}}$. Let *A* be the evaluated agent set, $F_{a}$ the predefined folds for agent *a*, $m_{a,f}$ the number of held-out known-positive glycans in fold *f*, and $r_{a,f,j}$ the one-indexed rank of held-out positive *j*. With ordered ranks $r_{a,f,(1)}\leq \cdots \leq r_{a,f,(m_{a,f})}$, the fold metrics are


$${\text{RR}}_{a,f}=\frac{1}{{\text{min}}_{j}r_{a,f,j}},\;\text{Recall}@5_{a,f}=\frac{1}{m_{a,f}}\sum_{j=1}^{m_{a,f}}I(r_{a,f,j}\leq 5),$$

$$AP_{\text{known},a,f}=\frac{1}{m_{a,f}}\sum_{j=1}^{m_{a,f}}\frac{j}{r_{a,f,(j)}}.$$

$AP_{\text{known}}$ treats all other candidates as nonrelevant and is average precision over the held-out known-positive relevance set. It is neither an upper nor a lower bound on complete-label average precision; higher values indicate better ranking. Aggregation follows the implementation exactly:


$$\text{MRR}=\frac{1}{\left|A\right|}\sum_{a\in A}\left[\frac{1}{\left|F_{a}\right|}\sum_{f\in F_{a}}{\text{RR}}_{a,f}\right].$$

Recall@5 and $AP_{\text{known}}$ use the same fold-within-agent and equal-agent averaging order. Percentile confidence intervals used 2000 bootstrap resamples of agents. Random-ranking and intact-vector feature-permutation controls used the same folds and candidates. The dated release retains its original 100-repeat control run; the final statistical audit repeats the same controls 1000 times with seed 42 to increase Monte Carlo resolution. Manuscript-facing null statistics use this high-resolution extension.

### 2.6 Direct representation comparisons

Direct comparisons comprised WURCS–SSV, WURCS–GCV, and SSV–GCV for MRR and Recall@5. Fold metrics were first averaged within each agent; paired differences were then computed across the same 159 agents. Confidence intervals used 10 000 paired bootstrap resamples of agents with seed 20260831. A deterministic two-sided sign-flip randomization test used 10 000 sign assignments, and Holm correction was applied across the six pair-by-metric comparisons. This family tests whether two representations differ across matched agents. It is separate from the six representation-versus-feature-permutation controls, which test whether each representation exceeds its own permuted-feature null.

The popularity scores were constructed from all 765 strictly joined relations across 227 agent identifiers. For each target agent *a*, every relation involving that agent was excluded from score construction, and each candidate glycan *g* was scored by its number of known-positive relations among the remaining non-target agents. Performance was then evaluated on the same 159 target agents, 697 evaluable relations, 44-glycan candidate universe, and 645 folds used for the representation analyses. Training-positive glycans were removed from each ranked candidate set, and equal scores were resolved by ascending GlyTouCan accession. Because accession ordering is reproducible but scientifically arbitrary within tied score groups, robustness was assessed with 1000 random tie-break seeds (20260901–20261900). For each agent-fold we recorded the number and maximum size of tied groups, whether the highest score was tied, whether a tied group crossed rank five, and the fraction of candidates involved in ties.

Two additional diagnostics tested annotation structure. First, the popularity control was recomputed after excluding every member of exact normalized-name duplicate groups and every member of groups sharing a recorded UniProt mediator; no approximate-name alias inference was used. Second, each held-out positive was stratified by its annotation degree computed only from non-target agents. Empirical tertiles, with integer ties assigned to the lower bin, defined low, middle, and high degree. WURCS and popularity were compared within strata using held-out rank, top-1 hit, Recall@5, reciprocal rank, and $AP_{\text{known}}$ contribution.

### 2.7 Prototype perturbation and sensitivity analyses

Prototype perturbation was estimable when a fold contained at least two training positives. Each training positive was removed in turn, the reference vector and candidate scores were recomputed, and the perturbed ranking was compared with the unperturbed ranking using Spearman score correlation, top-5 and top-10 Jaccard overlap, held-out-rank change, MRR and Recall@5 change, and reference-vector cosine similarity. For each leave-one-training-positive-out perturbation, signed held-out-rank changes were first averaged within the fold. The absolute value of this fold-mean signed change was then taken, and the resulting values were averaged across perturbations. Folds with one training positive were not perturbed and were reported as one-example reference vectors. Additional analyses varied minimum positive support (2, 3, and 5), GCV contact cutoff (4.0, 4.5, and 5.0 Å), and deposited model index (1–5).

### 2.8 Reproducibility and data release

Machine-readable evidence, exclusion, structure, feature, candidate, fold, per-fold result, sensitivity, and statistical tables are publicly available in the GlycoAudit repository. Automated consistency checks reconcile these tables, the manuscript-facing summaries, machine-readable source tables underlying the figures, and release checksums. Exact schemas, commands, and validation rules are documented in the Supplementary Information, available as supplementary data at *Bioinformatics Advances* online and in the public GlycoAudit repository. This provenance-first public release design follows the FAIR emphasis on findability, accessibility, interoperability, and reuse (Wilkinson *et al.* 2016, Watanabe *et al.* 2021).

## 3 Results

### 3.1 Strict structural joining removes most resolved glycans and enriches annotation density

GlyTouCan identifiers resolved for 1258 of 1263 SugarBind evidence records, representing 173 accessions. The strict structure contract retained 44 glycans, 801 records, and 765 canonical glycan–agent relations. Of these relations, 697 belonged to the 159 agents with at least two positive relations and entered the held-out-positive evaluation; the remaining 68 were associated with 68 agents lacking sufficient within-agent support for fold construction. Among the 129 resolved nonjoined glycans, 95 had no GlycoShape record, 29 lacked the exact requested accession branch, and five had a WURCS mismatch.

The joined glycans carried substantially denser annotation (Fig. 2). Median evidence records per glycan were 8.5 in the joined group and 2.0 in the resolved-nonjoined group; the standardized mean difference was 1.19. Median canonical relations were 7.5 and 2.0, respectively, with a standardized mean difference of 1.16. WURCS sequence length showed little standardized separation (−0.06). The strict join therefore selects an annotation-distinct evaluable domain rather than a neutral subset of all resolved SugarBind glycans.

**Figure 2:**
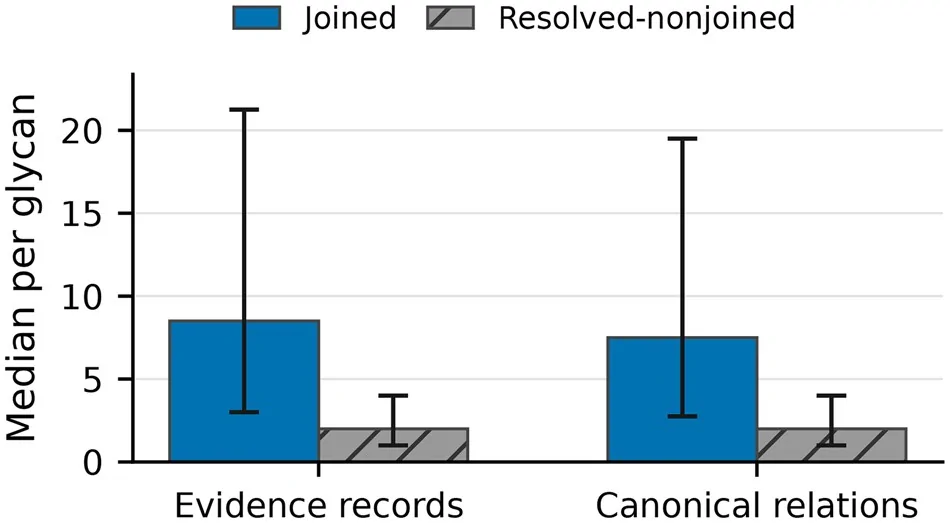
Annotation density in the strict joinable domain. Bars show medians for 44 joined and 129 resolved-nonjoined glycans; error bars span the first to third quartiles. Evidence records count source assertions per glycan, whereas canonical relations count distinct glycan–agent pairs. Complete standardized mean differences, bootstrap intervals, rank tests, and Holm-adjusted values are given in Table S1, available as supplementary data at *Bioinformatics Advances* online. Joined glycans carry markedly denser evidence and relation annotation.

### 3.2 Annotation degree changes apparent method ranking across metrics

The common evaluation contained 44 glycans, 159 agent identifiers, 697 known positive relations, and 645 agent-folds. WURCS achieved MRR 0.381, Recall@5 0.688, and $AP_{\text{known}}$ 0.373 (Fig. 3; Table 1). SSV achieved 0.258, 0.351, and 0.251, while GCV achieved 0.132, 0.159, and 0.128.

**Figure 3:**
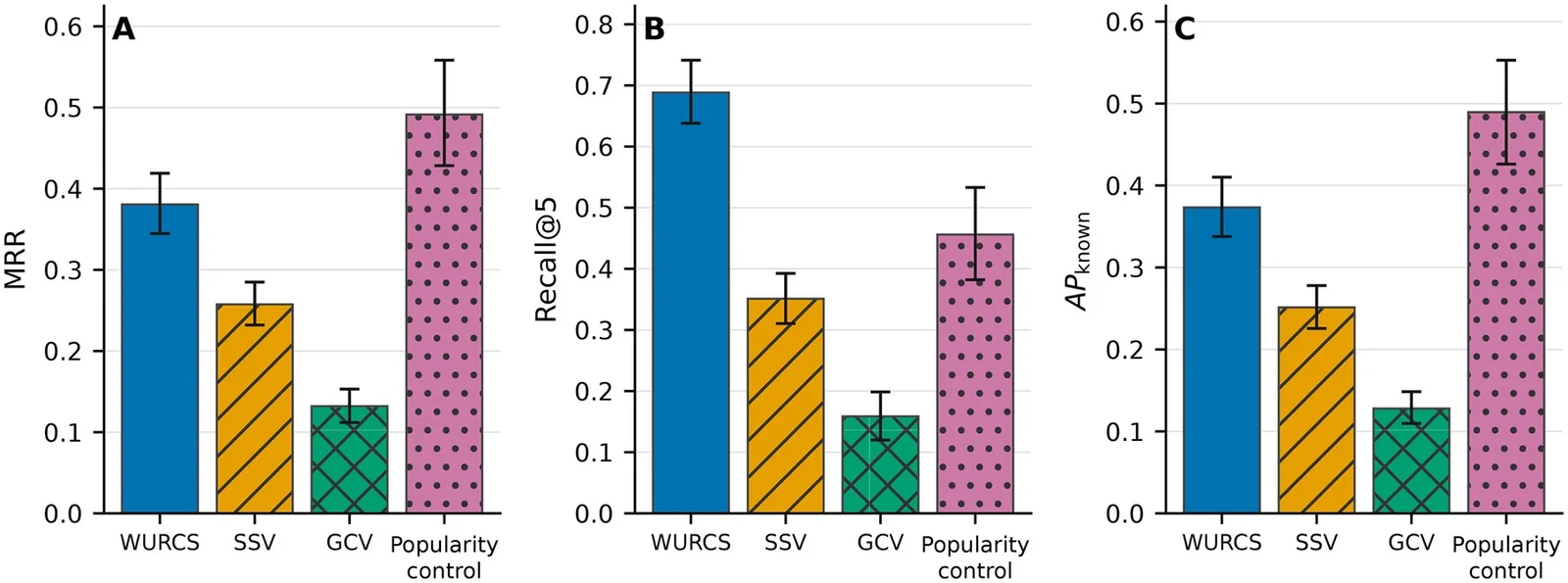
Held-out-positive ranking performance. Panels A–C show mean reciprocal rank (MRR), Recall@5, and $AP_{\text{known}}$ for WURCS, SSV, GCV at the 4.5 Å reference cutoff, and the leave-one-agent-out glycan-popularity control. Bars are equal-weighted means across 159 SugarBind agent identifiers after averaging the 645 folds within each agent; error bars are 95% percentile intervals from 2000 agent-bootstrap resamples. All methods are evaluated on the same 44-glycan fixed candidate set, 159 target agents, 697 evaluable positive relations, and 645 folds. The popularity control uses the same evaluation candidates and folds but constructs its leave-one-agent-out scores from the full 765-relation, 227-agent strictly joined reference pool as defined in Methods; it is not a molecular representation. Popularity is higher for MRR and $AP_{\text{known}}$, whereas WURCS is higher for Recall@5. Random tie-breaking and entity-exclusion sensitivities are reported in Tables S6–S8, available as supplementary data at *Bioinformatics Advances* online.

**Table 1:** Leakage-controlled held-out-positive ranking.^a^

Method or control	Mode	MRR	Recall@5	$AP_{\text{known}}$
**SSV**	Observed	0.258 (0.232–0.285)	0.351 (0.310–0.393)	0.251 (0.226–0.278)
**SSV**	Feature permutation	0.121±0.048	0.143±0.089	0.118±0.047
**GCV, 4.5 Å**	Observed	0.132 (0.112–0.153)	0.159 (0.120–0.199)	0.128 (0.110–0.148)
**GCV, 4.5 Å**	Feature permutation	0.116±0.047	0.133±0.084	0.113±0.046
**WURCS fold-TF–IDF**	Observed	0.381 (0.345–0.419)	0.688 (0.638–0.741)	0.373 (0.338–0.410)
**WURCS fold-TF–IDF**	Feature permutation	0.104±0.044	0.116±0.082	0.102±0.043
**Leave-one-agent-out glycan popularity**	Observed control	0.491 (0.428–0.558)	0.456 (0.382–0.533)	0.489 (0.426–0.553)
**All representations**	Random ranking	0.110±0.007	0.123±0.014	0.107±0.007

a Values are equal-weighted agent means with 95% agent-bootstrap intervals. Feature-permutation and random rows are means ± standard deviations over the 1000-repeat high-resolution extension. The popularity row is a leave-one-agent-out annotation-degree control, not a molecular representation.

The leave-one-agent-out glycan-popularity control achieved MRR 0.491 (0.428–0.558), Recall@5 0.456 (0.382–0.533), and $AP_{\text{known}}$ 0.489 (0.426–0.553). On matched agents, WURCS minus popularity was −0.111 (95% paired-bootstrap interval −0.169 to −0.054) for MRR and 0.232 (0.182 to 0.284) for Recall@5, and −0.116 (−0.174 to −0.060) for $AP_{\text{known}}$. Thus popularity exceeded WURCS for MRR and $AP_{\text{known}}$, whereas WURCS retrieved a larger fraction of held-out positives in the top five. No method dominated uniformly, and metric choice changed the apparent ranking.

All 645 agent-folds contained tied popularity scores; the median fold had 67.5% of candidates in tied groups, 287 folds tied at the highest score, and 76 had a tied group crossing the top-5 boundary. Nevertheless, the three headline orderings held for every one of 1000 seeded random tie-breaks. Mean random-tie values were 0.492 for MRR, 0.456 for Recall@5, and 0.490 for $AP_{\text{known}}$. Excluding the 21 evaluable identifiers in known exact-name or shared-UniProt duplicate groups left 138 agents and preserved the same reversal: popularity achieved 0.528 MRR, 0.496 Recall@5, and 0.526 $AP_{\text{known}}$, compared with WURCS values of 0.395, 0.727, and 0.389 on the same agents. These exclusions address only duplicate groups identifiable in the released metadata.

The degree-stratified diagnostic showed where the rankings diverged. Among low-degree held-out positives, WURCS achieved Recall@5 0.576 versus 0.299 for popularity and a top-1 rate of 0.318 versus 0.000. WURCS retained a Recall@5 advantage in the middle stratum, whereas popularity had higher reciprocal-rank and top-1 values in the middle and high strata; both methods reached Recall@5 1.000 in the high stratum. In the low-degree stratum, WURCS contributed 0.1157 to the overall equal-agent $AP_{\text{known}}$ mean, compared with 0.0352 for popularity. Popularity contributed more in the middle (0.1895 versus 0.0859) and high (0.2648 versus 0.1717) strata. Thus WURCS added its clearest relative value for less frequently annotated glycans, while popularity remained strong for well-annotated glycans (Tables S6–S9, available as supplementary data at *Bioinformatics Advances* online).

### 3.3 WURCS remains the strongest evaluated molecular representation under leakage control

Across matched agents, the WURCS–SSV difference was 0.123 (95% paired-bootstrap interval 0.092–0.154) for MRR and 0.337 (0.284–0.390) for Recall@5. WURCS–GCV differences were 0.249 (0.207–0.289) and 0.530 (0.458–0.601), and SSV–GCV differences were 0.125 (0.097–0.152) and 0.192 (0.148–0.236). All six sign-flip tests had Holm-adjusted *P* = .000600 within the direct-comparison family (Fig. S1 and Table S2, available as supplementary data at *Bioinformatics Advances* online).

The feature-permutation controls address a different null. With 1000 repeats in the final high-resolution audit, one-sided MRR probabilities were 0.0010 for WURCS, 0.0180 for SSV, and 0.2478 for GCV; Recall@5 probabilities were 0.0010, 0.0270, and 0.3217. Holm-adjusted values across this six-test family were 0.0060 for both WURCS metrics, 0.0719 and 0.0809 for SSV, and 0.4955 for both GCV metrics (Table S5, available as supplementary data at *Bioinformatics Advances* online). The original 100-repeat run remains part of the frozen release provenance, whereas the 1000-repeat extension supplies the final manuscript-facing null statistics.

### 3.4 Limited support and structural definitions constrain ranking stability

Thirty-six agents had exactly two positive relations, 89 had 3–5, 33 had 6–10, and one had >10. Raising minimum support from two to three and five reduced the evaluated population from 159 to 123 and 94 agents. Over these populations, WURCS MRR increased from 0.381 to 0.411 and 0.454, SSV from 0.258 to 0.299 and 0.336, and GCV changed from 0.132 to 0.144 and 0.126 (Fig. S2, available as supplementary data at *Bioinformatics Advances* online). Because the agent population changes with the threshold, these trends describe support-conditioned benchmark populations rather than within-agent learning curves.

Prototype perturbation was estimable for 573 folds from 123 agents in each representation. Removing one training positive produced mean candidate-score Spearman correlations of 0.970 for WURCS, 0.878 for SSV, and 0.873 for GCV; mean top-5 overlaps were 0.788, 0.643, and 0.587. The mean absolute fold-mean signed rank shifts were 1.73, 3.38, and 3.86 positions, respectively. Seventy-two of 645 primary folds (11.2%) had one training positive and were non-estimable for this perturbation; the 36 two-positive agents therefore contribute one-example reference vectors in both held-out directions.

#### 3.4.1 Coordinate and contact definitions

Across the five deposited GlycoShape model indices, SSV MRR ranged from 0.289 to 0.373 and GCV MRR from 0.155 to 0.270. GCV also varied with contact cutoff: MRR was 0.137 at 4.0 Å, 0.132 at 4.5 Å, and 0.273 at 5.0 Å, while Recall@5 was 0.167, 0.159, and 0.461 (Fig. S3, available as supplementary data at *Bioinformatics Advances* online). These analyses identify coordinate and contact-definition sensitivity that future local or interface-aware representations should address.

## 4 Discussion

The first interpretation layer is benchmark membership. The strict contract reduced 173 resolved accessions to a 44-glycan evaluable domain, and the joined subset carried much denser evidence and relation annotation than the 129 resolved-nonjoined glycans. Structural availability therefore affects more than sample size: it selects the evidence distribution on which performance is estimated. What can be evaluated is not neutral.

The second layer is annotation degree and metric dependence. Popularity winning MRR and $AP_{\text{known}}$ but losing Recall@5 is a ranking reversal, not a nuisance control. Random tie-breaking and known-duplicate exclusions preserved all three orderings, while degree stratification showed a WURCS $AP_{\text{known}}$ contribution advantage only among low-degree positives (0.1157 versus 0.0352); popularity contributed more in the middle and high strata. Together, these diagnostics show that annotation prevalence strongly shapes apparent ranking and that different metrics reward different parts of that structure, while leaving the underlying biological mechanisms unresolved.

The third layer concerns molecular representation. WURCS character *n*-grams, which encode residue and linkage syntax, exceeded the evaluated global SSV and GCV summaries on matched agents and were more stable to removal of a training positive. Within the released common protocol, WURCS is the strongest molecular representation among those tested; this benchmark-specific result does not imply field-wide representational superiority. SSV and GCV remain useful transparent diagnostics: their cutoff- and coordinate-model sensitivity motivates local, epitope-aware, interface-aware, multivalent, and ensemble-aware methods, consistent with recent graph, pocket, interface, and 3D glycan modeling work (Burkholz *et al.* 2021, Mohapatra *et al.* 2022, Bibekar *et al.* 2024, Carpenter *et al.* 2025, Chu *et al.* 2025, Thomès *et al.* 2025). A benchmark audit should precede model optimization.

### 4.1 Scope and interpretation

The present release estimates auditable held-out known-positive glycan–agent relation ranking within the strictly joined domain of 44 glycans from 173 resolved SugarBind accessions. Missing candidate relations remain unlabeled rather than confirmed negatives. CFG serves as provenance metadata because assay-resolved quantitative labels were not reconstructed, placing quantitative CFG and cross-source evaluation outside the current scope. Primary inference uses relation-level leakage-controlled, agent-stratified folds rather than scaffold-disjoint folds; the scaffold manifest supports the audit. Agent weighting follows SugarBind database identifiers; incomplete mediator mapping limits biological-entity-clustered inference and is documented in the released identifier audit.

The five deposited GlycoShape models support coordinate-sensitivity analysis rather than representing a validated complete solution-state or binding-competent ensemble. SSV and GCV are global summaries; richer representations are needed for local epitopes, protein interfaces, masking, and multivalent geometry. Folds with one training positive remain one-example reference vectors. These properties define the current benchmark scope and motivate future local, interface-aware, multivalent, and ensemble-aware representations.

## 5 Conclusion

GlycoAudit makes benchmark membership and evidence assumptions inspectable. Strict structural coverage selects an annotation-enriched domain, and annotation degree and metric choice can reverse apparent method rankings. WURCS is the strongest molecular representation tested here but is not uniformly superior to the non-molecular popularity control. The audited resource provides a defensible substrate for future local and interface-aware glycan models.

## Supplementary Material

vbag308_Supplementary_Data

## Data Availability

The source code, benchmark manifests, reproducibility scripts, and analysis artifacts associated with GlycoAudit are available at https://github.com/leochenminrui/GlycoAudit.
