# Supplementary material for: GlycoAudit: structural joinability and annotation degree shape glycan–agent relation ranking
Source: Bioinform Adv. 2026 Oct 20;6(1):vbag308. doi: 10.1093/bioadv/vbag308 (PMC13769440; doi:10.1093/bioadv/vbag308)
Supplement: Supplementary file 1 [file vbag308_supplementary_data.pdf]

Supporting Information

GlycoAudit: structural joinability and annotation degree shape glycan–agent relation ranking

Supplementary Note 1. Provenance and release scope

All current quantitative results use the dated `reconstruction_2026_08_04` release assembled from archived public snapshots. The historical correction record, including the withdrawal of the earlier self-retrieval, Consortium for Functional Glycomics (CFG) quantitative, cross-source, residualization, unspecified deep-learning, and scaffold-anomaly results, is retained in the point-by-point response and release audit documentation rather than repeated in the scientific narrative.

The release contains 1,263 SugarBind positive evidence records. Five source identifiers were unresolved; 173 unique GlyTouCan accessions were resolved; and 44 accessions satisfied the strict accession, branch, Protein Data Bank (PDB) annotation, and normalized Web3 Unique Representation of Carbohydrate Structures (WURCS) contract. The 765 canonical joined relations comprised 697 relations from 159 agents with sufficient support for fold construction and 68 relations from 68 one-positive agents that were excluded from evaluation. The 6,035-entry Consortium for Functional Glycomics (CFG) catalogue is metadata only because no assay-resolved quantitative label set was reconstructed. Automated consistency checks cover evidence and exclusion completeness, identifier and structure joins, fixed candidates, positive folds, aggregation, manuscript-facing values, and checksums. An internal revision-QC suite contained 43 passing tests; the public repository provides executable release-validation scripts. The reconstruction identifier remains `reconstruction_2026_08_04`; the manuscript-facing analysis package is `glycoaudit_manuscript_release_v1.0.0`. These are logical release identifiers rather than filesystem paths, and the corresponding materials are publicly available in the GlycoAudit repository. The frozen reconstruction is represented under `data/`, with its release manifest and checksums at `data/RELEASE_MANIFEST.json` and `data/CHECKSUMS.sha256`. The manuscript-facing package contains 1,108 files; the dated reconstruction retains its frozen 998-file inventory.

Supplementary Note 2. Data dictionary for structural features

The Structural Shape Vector (SSV) contains atom and residue counts, radius of gyration, maximum atomic separation, compactness, graph-derived branching and terminal-residue counts, and local atomic exposure. Features are computed per deposited coordinate model and released as five-model means and standard deviations.

The Glycan Contact Vector (GCV) contains contacts per residue, long-range contact counts, residue-neighbour contact summaries, variation in pairwise residue-centre distances, graph spectral gap, the relative balance of high- and low-degree residues, and maximum sequence separation among contact edges. For backward compatibility only, the machine field `contact_density` stores contacts per residue; `torsion_diversity` stores the coefficient of variation of pairwise residue-centre distances; and `core_periphery_ratio` stores the fraction of high-degree residues among the high- and low-degree groups. These aliases are software labels, not additional scientific quantities.

Each accepted glycan has five deposited models. SSV and GCV are computed per model, all per-

model values are retained, and glycan-level fields are unweighted means and standard deviations. GlycoShape metadata provide cluster populations, but the downloaded PDB multi-model files do not expose an auditable mapping between MODEL indices and metadata cluster identifiers; population-weighted aggregation was therefore not attempted.

The 4.5 Å GCV implementation default is treated as a reference cutoff, not a pre-result or mechanistically privileged threshold. The 4.0, 4.5, and 5.0 Å results are diagnostic sensitivity settings.

Supplementary Note 2A. Ranking metric definitions

The reported metrics are mean reciprocal rank (MRR), Recall@5, and known-positive average precision (AP_{known}). Let A be the evaluated agent set, F_a the predefined folds for agent a, $m_{a,f}$ the held-out known-positive count, and $r_{a,f,j}$ the one-indexed rank of held-out positive j. For ordered ranks $r_{a,f,(1)} \leq \cdots \leq r_{a,f,(m_{a,f})}$,

$$\mathrm{RR}_{a,f} = \frac{1}{\min_j r_{a,f,j}}, \quad \mathrm{Recall@5}_{a,f} = \frac{1}{m_{a,f}} \sum_{j=1}^{m_{a,f}} \mathbb{I}(r_{a,f,j} \leq 5),$$

$$AP_{\text{known},a,f} = \frac{1}{m_{a,f}} \sum_{j=1}^{m_{a,f}} \frac{j}{r_{a,f,(j)}}.$$

AP_{known} treats all other candidates as nonrelevant and is average precision over the held-out known-positive relevance set. It is neither an upper nor a lower bound on complete-label average precision; higher values indicate better ranking. MRR is the equal-agent aggregate of fold reciprocal rank. Folds are averaged within agent, and agent means are then weighted equally. Thus

$$\mathrm{MRR} = \frac{1}{|A|} \sum_{a \in A} \left[\frac{1}{|F_a|} \sum_{f \in F_a} \mathrm{RR}_{a,f} \right],$$

with the same aggregation order for Recall@5 and AP_{known}.

For the degree-stratified decomposition, the exact additive contribution of the kth ordered positive is

$$c_{a,f,k} = \frac{k}{m_{a,f} r_{a,f,(k)}}.$$

For stratum s, $C_{a,f,s}$ is the sum of these terms for positives in s, with zero assigned when fold f contains no positive in s. The reported contribution is

$$C_s = \frac{1}{|A|} \sum_{a \in A} \left[\frac{1}{|F_a|} \sum_{f \in F_a} C_{a,f,s} \right].$$

Consequently, the low-, middle-, and high-degree contributions sum exactly to the overall fold-within-agent, equal-agent AP_{known} mean for each method, up to floating-point tolerance.

Supplementary Table S1. Joinability-selection analysis

Forty-four joined glycans were compared with 129 resolved-nonjoined glycans. Five unresolved source identifiers were excluded. SMD is the standardized mean difference (joined minus nonjoined), and confidence interval (CI) is defined for the interval columns. Mean-difference intervals use 10,000 bootstrap resamples; rank-test values are two-sided Mann–Whitney tests and were Holm-corrected across four variables in the released table.

Variable	Joined median	Nonjoined median	SMD	Mean-difference 95% CI	Raw P	Holm P
WURCS sequence length	112.5	102.0	−0.064	−15.34 to 9.59	0.964	0.9638288509
Evidence records per glycan	8.5	2.0	1.193	8.27 to 21.84	3.25×10^{-8}	$1.3006482247 \times 10^{-7}$
Canonical relations per glycan	7.5	2.0	1.160	7.82 to 21.00	4.33×10^{-8}	$1.3006482247 \times 10^{-7}$
Agent identifiers per glycan	7.5	2.0	1.160	7.68 to 20.99	4.33×10^{-8}	$1.3006482247 \times 10^{-7}$

Supplementary Table S2. Direct paired representation comparisons

Fold metrics were averaged within agent before paired differences were computed across 159 agents. Intervals use 10,000 paired bootstrap resamples. Two-sided sign-flip tests used 10,000 assignments with seed 20260831; Holm adjustment covers the six pair-by-metric comparisons shown here.

Comparison	Metric	Mean difference	95% CI	Holm P
WURCS–SSV	MRR	0.123	0.092–0.154	0.000600
WURCS–SSV	Recall@5	0.337	0.284–0.390	0.000600
WURCS–GCV	MRR	0.249	0.207–0.289	0.000600
WURCS–GCV	Recall@5	0.530	0.458–0.601	0.000600
SSV–GCV	MRR	0.125	0.097–0.152	0.000600
SSV–GCV	Recall@5	0.192	0.148–0.236	0.000600

Supplementary Table S3. Prototype perturbation summary

Perturbation was estimable for 573 folds from 123 agents per representation. For each leave-one-training-positive-out perturbation, signed held-out-rank changes were first averaged within the fold; the absolute value of that fold mean was then taken, and these absolute fold-mean signed changes were averaged across perturbations. An additional 72 of 645 primary folds had one training positive and were designated one-example reference vectors rather than perturbed averages.

Representation	Score Spearman	Top-5 overlap	Reference cosine	Mean absolute fold-mean signed rank change
WURCS	0.970	0.788	0.979	1.73
SSV	0.878	0.643	0.912	3.38
GCV	0.873	0.587	0.848	3.86

Supporting Note 3. Relation to GlycanML, GlycoGym, and GlyContact

The comparison below uses the official ICLR 2025 GlycanML record and repository (`https://openreview.net/forum?id=owEQ0FTfVj`; `https://github.com/GlycanML/GlycanML`); the GlycoGym repository and archived benchmark release (`https://github.com/BojarLab/GlycoGym`; `https://doi.org/10.5281/zenodo.17313055`); and the GlyContact paper and official repository (`https://doi.org/10.1038/s41467-025-67590-y`; `https://github.com/BojarLab/glycontact`), accessed 1 September 2026. The repository's citation field remained "tbd", and the separately indexed ICLR 2026 submission was withdrawn; neither is represented here as an accepted conference paper. The earlier "ICLR 2025 Workshop" venue label was removed because an official publication record supporting that exact citation was not verified. GlycanML and GlycoGym are broader supervised glycan-learning benchmarks, whereas GlyContact retrieves and analyses three-dimensional (3D) glycan structures. None defines the same target, evidence semantics, candidate universe, representations, and folds as GlycoAudit; a direct numerical superiority comparison is therefore not valid.

Supplementary Table S4. Task-level resource comparability

Field	GlycoAudit	GlycanML	GlycoGym	GlyContact
Primary task	Held-out positive glycan–agent relation ranking	Multi-task supervised glycan learning	Seven supervised glycan-property and interaction tasks	Retrieval and analysis of 3D glycan structures
Unit of prediction or analysis	Glycan–agent relation	Task-dependent glycan or protein–glycan example	Task-dependent glycan, spectrum, interaction, or structural example	Glycan structure, conformer, residue, or structural feature
Identifier system	GlyTouCan and WURCS linked to SugarBind agent identifiers	Task-specific sequence and graph encodings	Task-specific benchmark tables and glycan encodings	Glycan sequences mapped to GlycoShape or user-supplied PDB structures
Evidence or label semantics	Source-positive relations; missing pairs remain unlabeled	Task-specific supervised labels	Task-specific supervised labels	No glycan–agent binding-label benchmark
Confirmed negatives available?	No	Task-dependent	Task-dependent	Not applicable
3D structure required?	Yes, for strict-domain membership	No	Task-dependent	Yes
Joinability explicitly audited?	Yes	No GlycoAudit-style audit	No GlycoAudit-style audit	No evidence-to-structure benchmark audit
Candidate universe released?	Yes	No matched relation-ranking universe	No matched relation-ranking universe	No
Split definitions released?	Yes	Task configurations and code are released	Dataset splits and builders are released	No
Exact task compatibility	Reference task	No	No	No
Direct numerical comparison valid?	Not applicable	No	No	No
Reason	—	Targets, labels, candidates, representations, and splits differ	Broader supervised tasks do not match the held-out relation-ranking protocol	Structural-analysis toolkit rather than a matched ranking benchmark

Supplementary Table S5. High-resolution null-control extension

The dated release contains the original 100-repeat null-control run. The final audit repeated the same random-ranking and intact-vector feature-permutation controls 1,000 times with seed 42, giving empirical resolution 1/1001. Values below are for the feature-permutation control; Holm adjustment covers the six representation-by-metric tests. The two runs test the same null and differ only in Monte Carlo resolution. Manuscript-facing values use the 1,000-repeat extension.

Representation	Metric	Raw P	Holm P	Null mean	Null SD
WURCS	MRR	0.000999	0.005994	0.104371	0.043692
WURCS	Recall@5	0.000999	0.005994	0.116472	0.082364
SSV	MRR	0.017982	0.071928	0.120519	0.047930
SSV	Recall@5	0.026973	0.080919	0.143196	0.089179
GCV	MRR	0.247752	0.495504	0.115835	0.047483
GCV	Recall@5	0.321678	0.495504	0.132890	0.084170

Supplementary Table S6. Leave-one-agent-out glycan-popularity control

The scoring reference pool comprised all 765 strictly joined relations across 227 agent identifiers. For each target agent, every relation involving that agent was excluded from score construction, and each glycan was scored using positive relations from the remaining non-target agents. Performance was evaluated on the separate population of 697 evaluable relations from 159 target agents across 645 folds, using the same fixed 44-glycan candidate universe as the molecular representations. Training positives were removed from the ranked candidates, and ties used ascending GlyTouCan accession. Values are equal-agent means and 95% agent-bootstrap intervals.

Control	MRR	Recall@5	AP_{known}
Leave-one-agent-out glycan popularity	0.491 (0.428–0.558)	0.456 (0.382–0.533)	0.489 (0.426–0.553)

Paired WURCS-minus-popularity differences were -0.111 (-0.169 to -0.054) for MRR and 0.232 (0.182 to 0.284) for Recall@5, and -0.116 (-0.174 to -0.060) for AP_{known}, using 10,000 paired agent-bootstrap resamples. No additional inferential P-value claim is made.

Ties occurred in all 645 folds. The largest tied group contained eight candidates; 287 folds tied at the highest score, 76 had a tied group crossing the top-5 boundary, and the median fraction of candidates in ties was 0.675. Across 1,000 deterministic random tie-break seeds, mean (SD; 2.5th–97.5th percentile; minimum–maximum) values were 0.49235 (0.00018; 0.49202–0.49269; 0.49168–0.49286) for MRR, 0.45597 (less than 2×10^{-16}; 0.45597–0.45597; 0.45597–0.45597) for Recall@5, and 0.49049 (0.00018; 0.49016–0.49084; 0.48984–0.49099) for AP_{known}. Popularity exceeded WURCS for MRR and AP_{known}, and WURCS exceeded popularity for Recall@5, in all 1,000 runs.

Supplementary Table S7. Known-duplicate exclusion sensitivity

Every member of a group sharing an exact normalized name or a recorded UniProt mediator identifier was excluded; no approximate-name alias inference was used. WURCS values below are means over the same remaining agents, while popularity scores were reconstructed after each exclusion.

Exclusion	Agents	MRR (P/W)	Recall@5 (P/W)	AP_{known} (P/W)
Shared UniProt groups (4 identifiers)	155	0.492/0.383	0.455/0.692	0.491/0.376
Exact-name groups (19 identifiers)	140	0.533/0.396	0.504/0.730	0.530/0.391
Union (21 identifiers)	138	0.528/0.395	0.496/0.727	0.526/0.389

P denotes popularity and W denotes WURCS. The WURCS-minus-popularity paired differences in the union analysis were −0.133 for MRR, 0.231 for Recall@5, and −0.136 for AP_{known}. These exclusions remove only duplicate groups identifiable from released metadata and do not establish complete biological-entity independence.

Supplementary Table S8. Popularity-stratified held-out-positive diagnostic

Degree was computed from positive relations among non-target agents only. Empirical tertiles over 697 distinct held-out known-positive relations defined low (0–40), middle (41–66), and high (72) degree; no evaluated positive had degree 67–71, and integer ties remained in the lower bin.

Degree stratum	Held-out positives, n	Contributing agents, n	Method	Mean rank	Top-1	Recall@5	RR	AP_{known} contribution to overall equal-agent mean
Low	246	97	WURCS	9.216	0.318	0.576	0.448	0.1157
Low	246	97	Popularity	11.761	0.000	0.299	0.126	0.0352
Middle	232	133	WURCS	5.947	0.023	0.707	0.225	0.0859
Middle	232	133	Popularity	3.534	0.470	0.534	0.576	0.1895
High	219	73	WURCS	1.849	0.352	1.000	0.643	0.1717
High	219	73	Popularity	1.023	0.977	1.000	0.989	0.2648

Held-out positives counts distinct target-agent–glycan relations. Contributing agents counts distinct target-agent identifiers represented in the stratum; an agent may contribute to multiple strata, so these counts may overlap and need not sum to 159. The sample-size columns are identical for WURCS and popularity. Mean rank, Top-1, Recall@5, and RR use the existing released diagnostic: held-out-positive values are averaged within each contributing agent and agents are weighted equally. The AP_{known} column is the exact additive contribution of positives in the stratum to the overall fold-within-agent, equal-agent mean defined in Supplementary Note 2A; the three stratum contributions sum to overall AP_{known} for each method.

In the low-degree stratum, WURCS contributed 0.1157 to the overall equal-agent AP_{known} mean, compared with 0.0352 for popularity. Popularity contributed more in the middle (0.1895 versus 0.0859) and high (0.2648 versus 0.1717) strata. WURCS also retained a Recall@5 advantage in the middle stratum, whereas popularity ranked the middle- and high-degree positives earlier by

reciprocal- rank measures. This supports the limited interpretation that WURCS has its clearest relative value for less frequently annotated glycans, while popularity remains strong for well-annotated glycans; it does not remove annotation-degree effects or establish a biological mechanism.

Supplementary Table S9. Agent-identifier metadata audit

Name groups use lowercase alphanumeric exact matching only; no similar-name or manual alias inference was performed. An external identifier means a released UniProt identifier for the recorded binding mediator, which is not available as a complete biological-entity key for all SugarBind agents.

Audit quantity	All source identifiers	Primary evaluation identifiers
Agent identifiers	386	159
With external UniProt mediator identifier	101	53
Not defensibly mappable from released metadata	285	106
Exact normalized-name duplicate groups	26	7
Maximum normalized-name group size	22	4
Shared external-identifier groups	6	2
Maximum shared external-identifier group size	12	2

Biological-entity-clustered inference is not estimable because external identifiers cover only a subset and identify recorded mediators rather than a uniform entity key. Equal weighting therefore remains at the database-identifier level and should not be interpreted as weighting independent biological entities.

Supplementary Figure S1. Paired representation differences

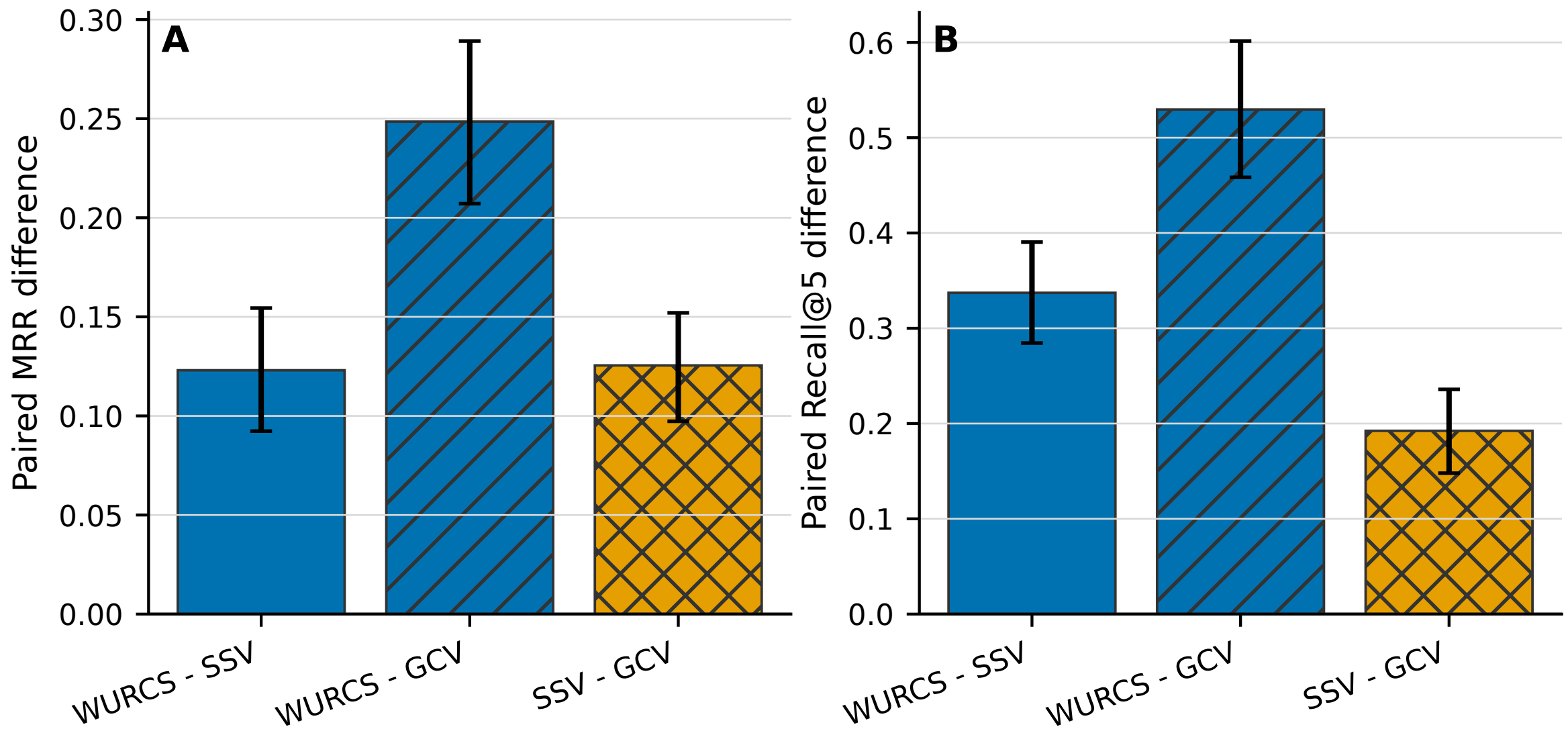

Panels A and B show paired MRR and Recall@5 differences for WURCS–SSV, WURCS–GCV, and SSV–GCV. Bars are mean differences across 159 matched agent identifiers after fold-to-agent aggregation; error bars are 95% paired-bootstrap intervals. The thin horizontal line denotes zero difference. Raw and Holm-adjusted sign-flip values are reported in Table S2. All comparisons favour the representation named first.

Alt text: Two vertical bar panels show three positive paired differences. WURCS minus GCV is largest in both panels; WURCS minus SSV and SSV minus GCV are smaller but remain above zero with their confidence intervals.

Supplementary Figure S2. Positive-support sensitivity

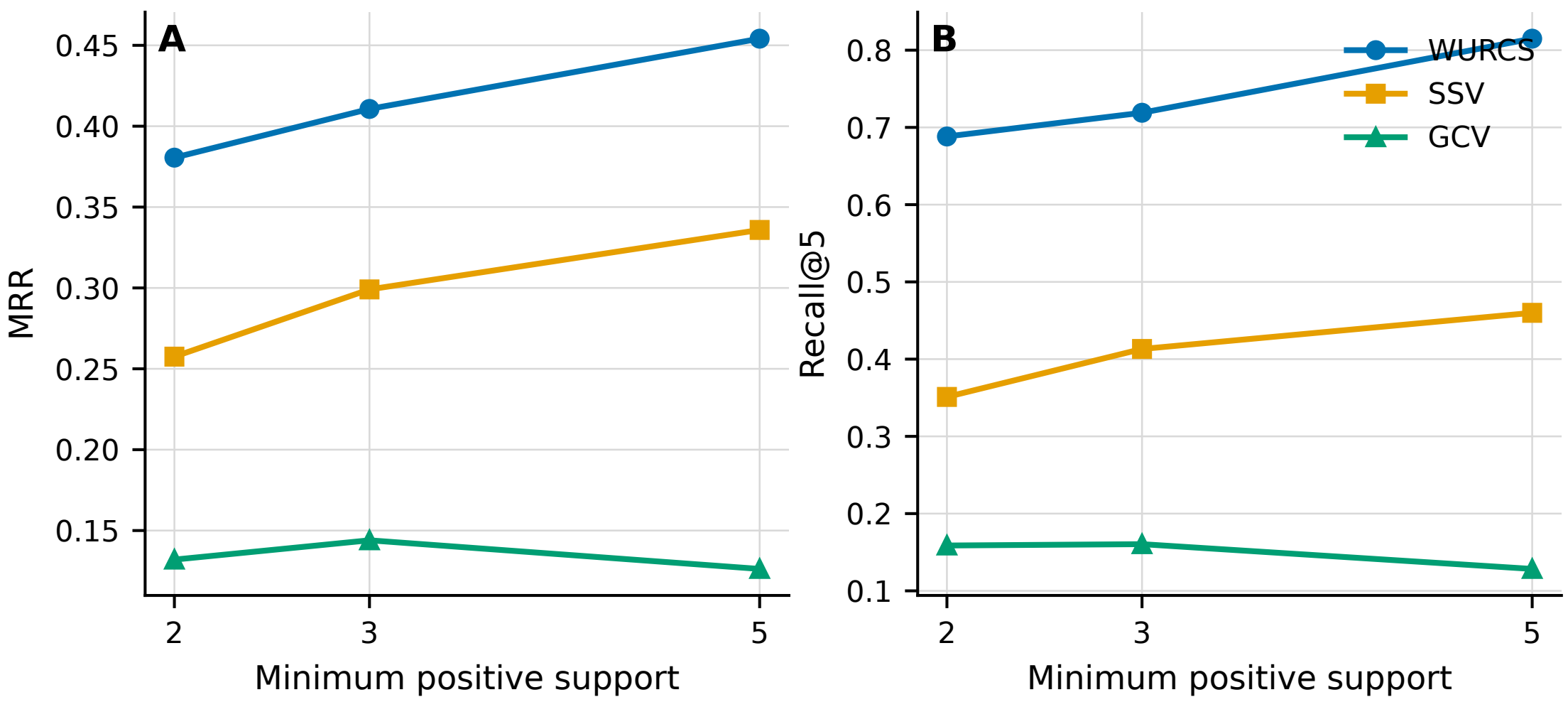

Panels A and B show MRR and Recall@5 as the minimum positive-support threshold is raised from 2 to 3 to 5. Lines represent WURCS, SSV, and GCV. The evaluated agent population changes from 159 to 123 to 94, so the curves compare nested benchmark populations rather than repeated estimates for a fixed agent set. WURCS remains highest at every threshold; GCV shows no monotonic improvement.

Alt text: Two line panels connect values at support thresholds 2, 3, and 5. WURCS is the upper line and rises with the threshold, SSV is intermediate and rises, and GCV remains low and falls at the highest threshold.

Supplementary Figure S3. Structural sensitivity

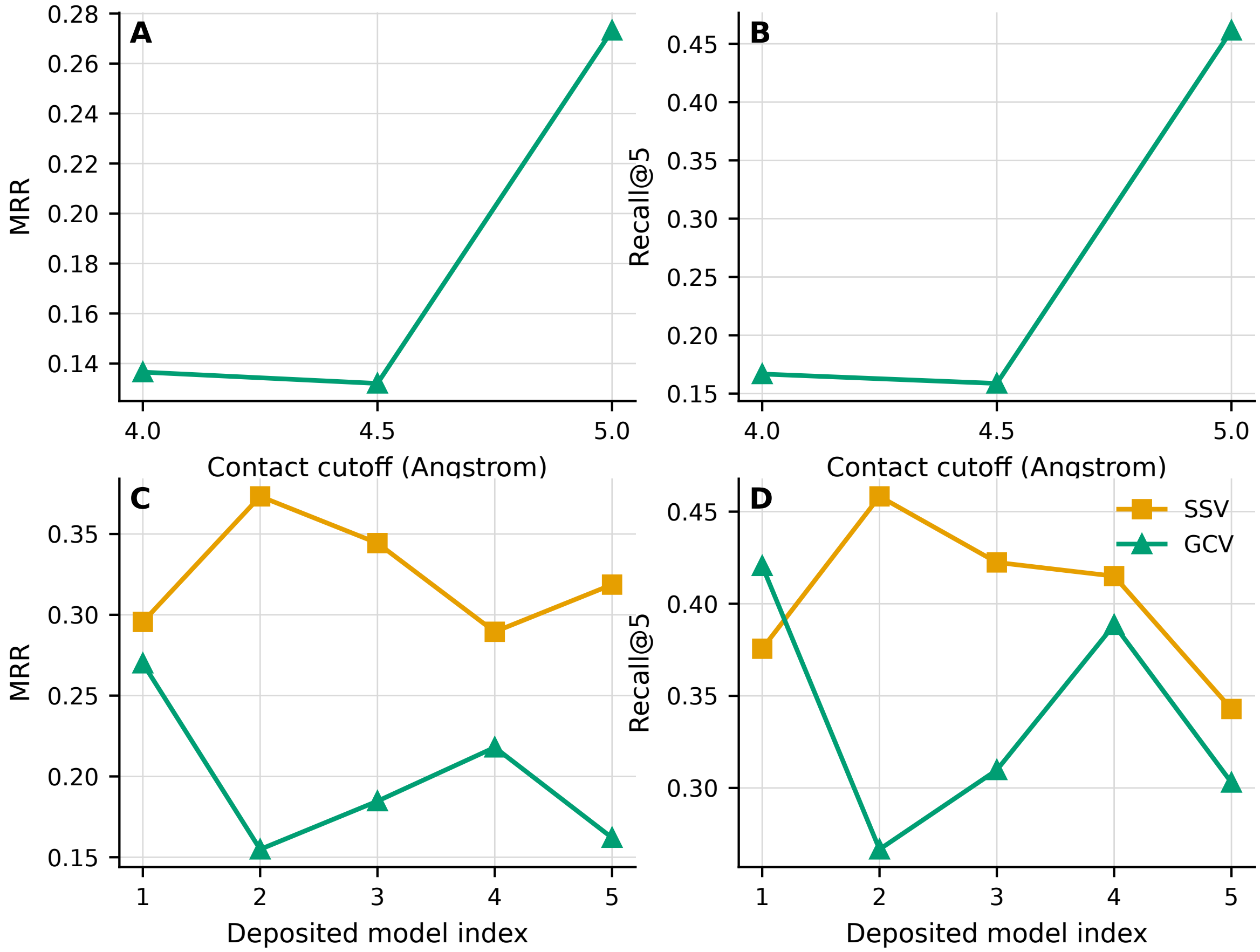

Panels A and B show GCV MRR and Recall@5 at contact cutoffs of 4.0, 4.5, and 5.0 Angstrom. Panels C and D show MRR and Recall@5 for SSV and GCV across deposited model indices 1–5. All analyses use 44 glycans and 159 agent identifiers with the same aggregation order as the primary evaluation. Model index is a coordinate- sensitivity analysis, not a time series or a validated ensemble trajectory. The 4.5-Angstrom setting is a reference implementation default rather than a mechanistically privileged cutoff. GCV changes sharply at 5.0 Angstrom, and both structural representations vary across coordinate models.

Alt text: The upper line panels show a sharp increase in GCV metrics at the 5.0-Angstrom contact cutoff. The lower panels show non-monotonic SSV and GCV values across model indices 1 through 5, with the ordering changing for Recall@5.

Supplementary artifact index

Machine-readable source tables underlying the manuscript and supplementary figures are publicly available under `results/`, including the joinability-selection, primary-summary, paired-comparison, popularity-control, prototype-stability, and structural-sensitivity outputs. Representative files are listed one per line to preserve clean path text:

```
results/joinability_selection_audit/continuous_feature_comparisons.csv
results/paired_representation_comparisons/paired_comparison_results.csv
results/primary_summary/primary_results.csv
results/primary_summary/minimum_support_sensitivity.csv
results/primary_summary/gcv_cutoff_sensitivity.csv
results/primary_summary/model_sensitivity.csv
```

Additional public analysis directories are:

```
results/popularity_baseline/
results/popularity_entity_exclusion_sensitivity/
results/popularity_stratified_diagnostic/
results/popularity_tie_sensitivity/
results/prototype_stability/
results/null_controls_high_resolution/
```

Release manifests, validation metadata, and checksums are provided in the repository root and under `data/`:

```
results/analysis_manifest.json
REFERENCE_RESULTS.json
CHECKSUMS.sha256
data/RELEASE_MANIFEST.json
data/CHECKSUMS.sha256
```
